# Minimal Invasive Endodontics: A Comprehensive Narrative Review

**DOI:** 10.7759/cureus.25984

**Published:** 2022-06-16

**Authors:** Jaydip Marvaniya, Kishan Agarwal, Dhaval N Mehta, Nirav Parmar, Ritwik Shyamal, Jenee Patel

**Affiliations:** 1 Department of Conservative Dentistry and Endodontics, Teerthanker Mahaveer Dental College and Research Centre, Moradabad, IND; 2 Department of Oral Medicine and Radiology, Narsinbhai Patel Dental College and Hospital, Sankalchand Patel University, Visnagar, IND; 3 Department of Conservative Dentistry and Endodontics, Faculty of Dental Science, Dharmsinh Desai University, Nadiad, IND; 4 Department of Conservative Dentistry and Endodontics, Private Practitioner, Contai, IND; 5 Dentistry, Private Practitioner, Junagadh, IND

**Keywords:** minimally invasive endodontics, peri-cervical dentin, soffit, root canal therapy, dentin

## Abstract

It is the aim of any surgical procedure to restore the tooth to its normal form and function, as well as to restore the tooth's appearance when it is appropriate to do so. One of the primary purposes of endodontic therapy is to clear out the root canal system of germs, pulpal remains, and other foreign matter. A tooth's biomechanical properties have to be compromised in order to achieve this goal; hence the tooth has a poor prognosis for restorative success. The remaining dental structure and restorations have a significant impact on the long-term viability of an endodontically treated tooth. Minimally invasive endodontics (MIE) is an endodontic technique that aims to maintain as much of the healthy coronal, cervical, and radicular tooth structure as possible. Access opening, root canal cleaning and shaping, and surgical endodontics are all possible applications for MIE in endodontic treatment. The objective of new-age endodontics is minimum intervention, and this review article examines a variety of methods that may be combined at each level of endodontics to reach this goal. A favorable outcome with minimally invasive treatment may be achieved while preserving the tooth's natural structure with careful case selection.

## Introduction and background

A successful root canal procedure increases the likelihood that the tooth will continue to function normally within the mouth for a significant amount of time [1]. After endodontic treatment, the lifetime of a tooth is dependent on the amount of residual tissue and healing. Endodontic outcomes are also affected by factors such as the quality of the root canal filling as well as the structural integrity of the tooth after the root canal preparation. Dentine behavior and configuration throughout age and function are presently the focus of current research efforts in this field [2]. Although a more conservative approach is being promoted in the present day, this has been made feasible by many new technologies and processes. The significance of preservation and a cautious therapeutic strategy cannot be understated [3]. This innovative kind of root canal therapy, known as minimally invasive endodontics (MIE), takes a different approach that focuses on minimizing structural alterations after treatment [2]. Treatment and prevention of pulpal diseases and apical periodontitis, as well as preserving as much good tissue as possible, are all part of the MIE method, which is safe, accurate, and time-saving [4]. Endodontic procedures are becoming less invasive because of the development of new materials and technology, such as cone beam computed tomography (CBCT) scans, advanced microscopes, unique access designs, and inventive access cavities [5]. Root canal anatomy is one of the most important aspects of healthy living with good dental health. In this respect, the field of endodontics, which has entered a new era, contributes to the progressive development of dental healthcare. This is effective for the development of the minimally invasive treatment, which assists scientists, physicians, and dentists to detect the structure of teeth, diagnosing the issues from teeth, and a careful analysis of the tooth's natural structure. This is necessary to provide effective treatment for the development of tooth conditions and provide human beings with good dental health, which can result in healthy living. In that case, root canal anatomy is a part of endodontics, which provides a new lease of life to decayed teeth and develops a balance in tooth health management.

## Review

Preserving structural integrity

Some of the ideas in the way dentistry has been practiced over the past century have recently undergone a paradigm shift. The tooth's residual structural integrity is a critical component in determining prognosis in terms of future function after restoration. The purpose of all restorative therapies, notably in endodontics, is to maintain strength and stiffness that resists structural deformation. For example, extension for prevention is no longer found to be widely accepted, as it requires removal of the sound and intact tooth structure for only a potential chance of a future benefit. Understanding the biomechanical behavior of dentin as the weakest link in any restorative system is essential if we are to avoid causing more harm to it [5,6].

According to the in-depth research by Silva et al., there have been cases when endodontically treated teeth were extracted because of inadequate repair of the dental structure [7]. Therefore, to boost the prognosis of endodontically treated teeth, healthy dental material must be retained. Six multiple studies demonstrate that a tooth with extensive enamel and dentin loss performs much worse than an undamaged tooth when it comes to the capacity to bear occlusal and functional forces. Reduced access preparation diameter by half resulted in the operator removing four times less tooth structure in MIE compared to a standard preparation. A higher density of dentin boosted the tooth's tensile and fracture strength, making it more resistant to breakage.

Based on logical reasoning, the preservation of maximal dentine mass during access cavity preparation and root canal shaping is of utmost importance; to date, no manufactured substance can effectively compensate for the lost dentinal tissue because of its unique features and traits [7].

Endodontic therapy is a common dental technique used to repair teeth whose pulp tissues have become permanently irritated or decaying as a result of caries or dental trauma. This treatment, which comprises mechanical and chemical root canal preparation, may affect numerous mechanical and physical aspects of the tooth structure [8]. Endodontic therapy can also affect the lifespan of endodontic treatment, tooth rehabilitation, and biomechanics during oral function. Several factors and clinical judgments must be followed while recovering endodontically treated teeth. The choice of fiberglass posts and restorative materials is influenced by several criteria, including the availability and value of surviving dental structure, the existence of a ferrule, the duration of the post cementation, and the ultimate coronal restoration.

Biomechanics refers to the study of biological structure, as well as its function, using the principles of engineering mechanics. Endodontically treated teeth (ETT) are effective for structural management of biomechanics, which play a vital role to increase nutritive tissues in dental health. Furthermore, it focuses on the development of the dental tissues and restorative tooth structure. Endodontic therapy focuses on the good health of the dental areas, and biomechanics focuses on the natural dental system of chewing and assisting the human body with digestion. In this concern, biomechanics plays a vital role in resisting fracture, and also lessens the pressure on weakened, as well as decaying, teeth. In other words, ETT promotes better handling of dental tissues so that an ideal prognosis can be achieved. The biomechanics of dentin and its behavior are altered at three levels because of endodontic treatment, which are dentin macro and micro-structure, the composition of tissues, and an all-over structure of tooth [9].

Biomechanics and biomechanical preparation, in particular for root canal treatment, are useful for the development of cavity-related disorders. Root canal therapy is a treatment sequence for a damaged tooth's pulp that concludes in the eradication of infection and the protection of the remediated tooth against additional microbial invasion. The term "root canal therapy" refers to this treatment sequence. One of the most significant parts is access cavity preparation, with the main goal of identifying root canal openings for further biomechanics preparedness and complete removal of the "root canal system" [10]. As a result, great access cavity design is critical for high-quality endodontic treatment. The notion of "extension for prevention" simplifies treatment operations, however, this also eliminates crucial dentin at the cervical area, leaving natural teeth biomechanically impaired following endodontic therapy.

MIE fosters an iatrogenic root dentin removal system, which influences radicular stress distribution on teeth. This also experimented with the root dental system to analyze the root volume, on radicular bending structure. A load of fracture and stress distribution is necessary for the management of the dental diagnosis and analysis of the root health of teeth. In other words, this focuses on the management of mechanical integrity of ETT or endodontically treated teeth [11].

Biomechanics of dentine

A vital cellular component, the dentine houses the cellular processes of the pulp-dwelling cells known as odontoblasts [12]. Biomechanics is the branch of engineering mechanics that deals with the study of biological structures and their functions in terms of mechanical principles. Natural collagen provides strength, whereas the inorganic component of dentin can withstand the hardness and high compressive properties of dentin because of its water-based nature. Tensile stress in compression is greater than tensile stress in tension. The small pulpal environment and fluid in dentinal tubules all work together to prevent cracks in the dentin from occurring [13]. Endodontic therapy may cause a decrease in dentin water content, which might lead to dentin tissue contraction and the formation of cracks and fractures that could lead to tooth breaking [14].

During endodontic treatment, tissue composition, dentin macrostructure, and total tooth structure are all altered. With age, our dentin becomes more brittle and less elastic due to physiological and pathological processes. This leads to a decline in the mechanical properties of our teeth. There must be an appropriate equilibrium between the rigidity of dentin (mineralization) and the toughness (elasticity) of the tissue (collagen and hydration water) [13].

When evaluating the long-term efficacy of endodontic treatment, the decreased fracture resistance of root canal-treated teeth remains a serious concern. According to Reeh et al., the structural integrity of teeth treated with endodontics is a critical factor in their long-term survival (1989). Also, as per Mauger et al. (1993), and Willershausen et al. (2008), important for long-term root canal treatment success is knowledge of root canal morphology, chemomechanical preparation processes, and cavity designs. As per Tang et al. (2010), pericervical dentine and structural integrity of treated teeth are important considerations in determining the long-term outlook of an individual tooth's root canal treatment. Endodontically treated teeth with preserved hard tissue are more resistant to breaking and have lower stress concentrations, especially in the cervical region [15]. A minimally invasive method and various preparatory equipment should be used to properly preserve dentine during endodontic treatment, even if the loss of tooth structure is not the primary cause of reduced fracture resistance in endodontically treated teeth [16].

With this objective in mind, pericervical dentin preservation has been advocated for in endodontics using the MIE method maintaining dentin, which acts as a stress-transmitting conduit, may increase the strength of the tooth. Removal of dental hard tissues such as the oblique ridges, peri-cervical dentin, and the thinning of marginal surfaces for clinical convenience may increase the risk of tooth breakage, they developed this procedure [17].

Minimally invasive endodontics: from conservation to survival

When an adult tooth has been found to have irreversible pulpitis or apical periodontitis, root canal therapy is the only option for saving it. As a consequence of the procedure, the treated tooth loses its hard tissue, leaving it more prone to fracture [18]. With minimum tooth structural loss, MIE is a clinical strategy for the practice of endodontic procedures and instruments. The root canal system must be adequately cleaned, shaped, and filled without compromising a significant amount of occlusal enamel and dentin in the crown and roots. This is the core principle. Endodontics pioneers Clark and Khademi have been credited with the development of minimally invasive access cavities [19]. The occlusal and cervical portions of the tooth are less stressed according to the Consortium for Educational Communication (CEC) design, which preserves more of the coronal tooth structure (Yuan et al., 2016). As a result, the tooth's ability to withstand masticatory stresses may be enhanced by a minimal canal preparation with a reduced taper [20].

For more than a decade, regenerative endodontic procedures have been discussed as a paradigm change in the diagnosis of immature necrotic permanent teeth due to their ability to allow root maturation, resulting in increased fracture resistance, as well as the possibility for regeneration of vital intracanal tissues. Simultaneously, minimally invasive root canal therapy is a growing idea with the primary goal of preserving tooth structure. Because of their ability to retain the natural tooth structure, regeneration and minimally invasive endodontics might be seen as two innovative disciplines with a single purpose. By supporting a new combined notion of minimally invasive regenerative endodontic procedures (MIREP), novel biomimetic cell-friendly disinfection chemicals and techniques may be feasible. This enhances the treatment and outcome of clinical treatment [21]. It also includes a customized disinfection or regeneration program for minimally invasive endodontics in terms of dental health.

Endodontic therapy's primary purpose is to prolong the life of a functioning tooth by preventing and/or treating apical periodontitis. However, good endodontic results are dependent on a variety of circumstances, including the "structural integrity" of the teeth during root canal preparation and the eventual restoration's quality. Endodontics is constantly evolving as new procedures and technological improvements are introduced. Currently, "minimally invasive endodontics" is a great approach for endodontic therapy, with irreversible restorative options deferred until after the procedure. Various materials and approaches have been developed and/or suggested to extend the life of endodontic treatments while retaining enough root strength and balancing physiological, mechanical, adhesion, operational, and cosmetic characteristics. This focuses on instrumentation techniques for the development of endodontics. Some of the techniques are multiphase techniques for risk identification regarding microleakage, vital pulp therapy or "endolight" (which is a biology-driven treatment protocol), and current fabrication technique. These are quite effective with computer-aided manufacturing or design to maximize tissue preservation and develop dental health. 

"Minimal intervention dentistry" is the current approach for the development of the technology-based intervention for the development of the dental healthcare aspect. This develops endodontics with a technological revolution in the management of instruments, materials, optics, and computer-based systems [22]. This fosters long-term success and fulfillment of biological goals; again, this is effective for the development of tooth structure with the development of the "minimally invasive endodontic" philosophy, which is based on maximum preservation of healthy coronal, radical, and cervical tooth structure at the time of endodontic therapy. This "minimal intervention dentistry" is effective for the development of the clinical decision-making processes, with the perception of "saving dentine", with developed technically well-treated canals [23]. This develops clinical rules and policies for the development of infection management, and fracture management. In other words, the development of clinical technology, especially computer-based systems, is highly effective for the development of endodontic treatment processes.

Minimally invasive access strategies

The complexity of root canals and human pulpal systems make endodontic treatment a challenging endeavor. To begin, the root canal system should be accessed, shaped, and cleaned in such a manner that the root canal region may be filled efficiently and completely while leaving the tooth with sufficient strength to function. Tooth fracture toughness has been reduced by using straight-line access to the orifice in standard access cavity designs [1]. It is easier and more comfortable to do minimally invasive procedures now than it was in the past because of advances in illumination and magnification technology [24].

As a part of MIE, attention has been paid to the tooth's peri-cervical region (about four millimeters above and below the alveolar crest). Dental health is dependent on protecting the peri cervical dentine (PCD), particularly in the molars, for long-term viability and optimal function. As a result of this new policy, dentists are no longer encouraged to utilize round burs or Gates-Glidden burs. To avoid structural flexure and eventual collapse, the access and coronal canal space must be protected from gouging. Dentin conservation and protection above and below the PCD are made possible by the dentist's use of a more effective and well-proven technique for reinforcing an endodontically treated tooth. In these critical locations, no man-made substance or technology can make up for missing tooth structure [4, 25].

There have been several modern designs tested to attain the best possible level of fracture resistance [1]. These include parts such as conservative endodontic access cavity, ninja endodontic access cavity, orifice-directed dentin conservation access cavity, incisal access, Cala Lilly enamel preparation, and micro-guided endo access. It has been claimed that the modern implantation of a continuous-flow left ventricular assist device (CF-LVAD) can be performed using less invasive techniques and with alternative tactics as well as their implementation. This strategic perspective is highly convenient for the patient who has a massive risk of heart failure during the time of endodontic access [26].

The above-mentioned strategy is all about the beneficial perspective of endodontics. However, the disadvantage of this process is further associated with thoracotomy incisions. Minimally invasive endodontics is an alternative to structural alteration due to root canal therapy and a strategic representation of numerous paradigms that constitute a substantial challenge to dental correction. Several preparations are associated with the minimally invasive strategy for measuring the concept for future betterment.

The problems of endodontic therapy are related to the intricacy of the individuality of the root canal system of the human pulpal system [27]. Throughout this therapy, steps should be followed for the maintenance of improved dental orientation, as well as to shape, access, and clean the canals in an effective manner. The exposal of the single teeth could be demonstrated in single-line access and this exactly is taught to the dental students. The smallest dimension and the cavity exposure become stronger through this process and its success rate becomes very low for the patient hence the strategic implementation of the invasive strategy is required to structure the doctrinaire access paradigm more concisely [27]. The major strategic implementation that is absolutely associated with an invasive strategy is about shaping out of the root canal space more conveniently by allowing internal compressive force for the obstruction, to manage the required consideration that is associated with the invasive endodontics disinfectant and other cavity design, to create a restoration strategy for minimal invasion and the absolute protection by allowing the best possible positive report and not dependency as it is required to maintain the possibility over the root strengthening system 

Shaping the root canal space

In order to withstand obturation's internal compressive stresses, root canal forms must be strong enough to hold softened and compressible filler materials, and should also be strong enough to withstand mastication.

Because of this, endodontists nowadays tend to follow one of two basic patterns when it comes to shaping techniques. Larger diameters and limited tapers in the canal form are thought to impair the root structure and reduce the ability to regulate obturation factor in treatment by a substantial number of dentists. The preparation that promotes resistant forms and an apical seal that is tight is supported by these methods. A cautious approach to developing a suitable shape for disinfection is supported by these methods.

A substantial body of data, on the other hand, suggests wider apical canals are beneficial for sculpting the apical canal wall, flushing debris, increasing terminal irrigation depth, and lowering bacterial contagion in the system over time.

The purpose of shaping the root canal is all about facilitating the area as clean in order to fill that area with obturating material for the future. The cleaning process is mandatory for the root canal procedure because the bacteria is already affecting the area and after the proper implantation of the root canal the gum should be protected; as it is known to all, oral health is required. It is a myth that a root canal can change the shape of the face but it also bears mentioning that with the procurement of good technology this perspective can easily be mitigated. Therefore, the natural teeth of the roots should be preserved that help to manage the total oral health of the patient for further maintenance of preventing bone loss, underlying jaw healthy, and a changing face shape. The infected root pulp never heals on its own hence, it is required for the dentist to be very specific during the time of the root canal procedure. 

An abundance of research demonstrates the superior centering and dentin preservation provided by nickel-titanium technology alone or in conjunction with stainless steel equipment [4].

Cervical area preservation

The terms peri-cervical dentin (PCD) and soffit were first introduced to the endodontic world by Clark and Khademi. As the name suggests, PCD describes the dentin located closest to the alveolar crest. The essential zone: In order for the PCD to transmit pressure from the occlusal table into the root, it relies on this four millimeters coronal and four millimeters apical [28,29].

Dentin soffit protects the peri-cervical dentin by covering the whole coronal section of the pulp chamber [30]. As the name suggests, the ferrule is a metal band that encircles all of the dental structure surrounding the cervical region. “Ferrule is defined as a circular area of axial dentin that extends from the preparation edge of a tooth to the cervical section of the tooth. The axial wall of the tooth refers to the dentin that surrounds the tooth's cervical area and is covered by the axial wall of the crown [9]. Traditional ferrule, dentin girth, and occlusal convergence are all components of this system.”

A study by Allen et al. suggests that traditional endodontic cavities (TECs) may be more susceptible to fracture than minimally invasive approaches. According to certain research, root fracture may occur if the pericervical area is removed too much tissue during the construction of the access cavity. It was revealed that the tension in the crown and cervical areas was less uniformly distributed when a minimally invasive approach was applied [31].

This aspect has to be evaluated with the best possible approach to the endodontic treatment of teeth after the management of root canal preparation and cervical preflaring. This perspective can easily help to promote the concept of root canal volume assessment and its remaining dentin part after and before cervical preflaring. Canal preparation and the part of cervical preflaring are always assisted to reduce the dentin thickness and increase the proper volume of the canal [32]. The efficiency of Gates-Glidden burs helps to reduce the threat of fracture during the time of endodontic treatment of the teeth. The assumption process that is associated with cervical preflaring is associated with the importance of the previously used root canal process for individuals to minimize the threat of operational risk through the endodontic process. The accuracy of apical diameter and working length become established more conveniently with the help of cervical preflaring and it manages the preparation of canal diameter by assuring non-exceeds process for root width. The instruments that effectively help to demonstrate the total process of cervical area preservation are the Largo, Gates-Glidden, NiTi instruments, and LA Axxess burs. Most researchers prefer not to use these burs during the operation and cervical implementation by crown down approach. Fracture resistance efficiency and cement junction process for the cervical preflaring is the major requirement that this endodontic process has to be aware of by professionals for future advancement in the scientific institute. The uses of the software within this cervical performing measurement are CTan v.1.12 software that helps to maintain the dentin thickness [33]. The total perspective of the cervical preflaring method is all about the non-invasive method and micro CT scanning is used for its sample selection initiative. Implementation of the cervical preflaring instruments is helpful to resist the fracture of the teeth and provide efficiency within the health management process for the individual. The use of the endodontic process easily assists the perception of the cervical preflaring by evaluating the dentin thickness of the teeth. In terms of illustrating the process of cervical preparation the use of Gates Glidden is more convenient and proceeds with a positive result [34]. The total structure of the eternal ad external repair process is somewhat impossible without the help of an endodontic system. External cervical resorption (ECR) provides the dependency over the accessibility and the nature of the ECR process with more extensive theory and treatment.

Disinfection and other considerations in minimally invasive endodontics

Instrumentation and disinfection are critical to root canal treatment's success. At this point, it is necessary to clean off any remaining dirt and smear layer. There is a strong correlation between root canal infection and treatment failure, as shown by the large majority of patients who do not respond well to regenerative endodontic [20]. Research by Plotino and colleagues found that an instrument with a 25-size bore provided a better canal surface in the root canal's apical third [35].

Root canals are unlikely to be fully free of bio-burden with existing cleaning and shaping processes because of the present level of technology. In light of the shrinking size of apical preparations, scientists are investigating novel strategies for increasing irrigation effectiveness. “It is possible that future root canal preparation methods will have to balance the ability to disinfect and iatrogenic injury with better debridement and disinfection capabilities [4].

Microbial elimination is the major goal of endodontic therapy with the required technological assessment and indirect manipulation of the dentin. For achieving the goal of the treating clinician has to become less simultaneously invasive. Teeth are an important part of the human being and to save this treasure it is the major responsibility of the individual by balancing those in an appropriate routine check-up. The most convenient advantage is that MIE is associated with invasive leads for managing the rate of procedural errors (missing canals, perforation, ledging, or instrument separation). Benefits become further provide poor outcomes with its outweighed measurements [17]. Hence it is required to be conservative in order to help standardize the microsurgical access preparation that helps to save enamel or dentin at a high rise cost. In the addition to the implementation of the new innovation, in this aspect use of different instruments or tools could be a great sign of success without any potential compromise. Without any unnecessary flaring MIE is capable to preserve the dentin by the synchronized hydraulic condensation. No doubt the efficiency and the merit of the MIE is incredible during the use of potential promotion of disinfectant and other consideration, but the strive of MIE is associated with the negative consequences of preparation for micro-access [21]. Minimally invasive techniques and technology are the proper rationales for endodontics. The term “over-instrumenting’ has helped the probability of a technical catastrophic breakdown and by aligning the concept of the MIE technique, thinking and the preferred outcome can be evaluated. In terms of biological success and sustainability maintenance, the MIE has to be taken forward by clinicians without the sanctification of the relevant tissues in terms of three particular aspects: long-term survivability measurement, progression for the biological success, and critical evaluation of the root canal process.

Restoration strategies and their importance in minimal invasion

In addition, root canal filling quality is assessed by the sealant's resistance to leaking and its stiffness. For teeth with minimally invasive access cavities, there are bigger gaps in the fillings of the canals, says Rover et al [36]. As seen by the oval form of the mandibular incidor, a little access cavity is insufficient to seal the canal.

Filling or crown should be used to repair the tooth after root canal therapy. Depending on the degree of hard tissue loss, a variety of repair options may be investigated. If at least 2mm of pulp chamber material remains after endodontic treatment, an endo-crown design may be used to reconstruct teeth with severe structural material loss. In comparison to post and core restorations, which might harm the root, endocrown restorations are less invasive [31].

The restoration strategies for the implementation of the root canal process can be developed by the clinician are appropriate. During this time, the process that could be more convenient is tooth location, number of proximal contacts, definitive restoration timing, tooth volume, and the presence of cracks, which can influence the process of restoration and tooth survival. The conservative endodontic-restorative method helps to strategies the total structure in a scientific way. The restoration process can be followed with several terms. When it comes to clinical success, the treatment of root canal in vivo studies is of more importance as the practitioner can assess the response in the patients themselves [36]. The strategies associated with the restoration failure are often associated with the failure and fractural implementation due to the extraction of the teeth. The definitive restoration process is the efficient outcome for the root-filled teeth and the choice of the final restoration process is a matter of concern of a proper consideration that assists to guide the patient with a proper decision-making initiative. Strategies for the teeth filling and its restoration process are as follows as in the root canal system: it is required to prevent the microbial leakage, form of restoration, adequate contact points, and occlusal stability for maintaining the development of the neighboring teeth, the functional restoration process, residual teeth, and their structure is required to protect against hard tissue fracture and loss development [37], marginal periodontal tissues and the maintenance of their health, and management for the optimal aesthetics.

Moreover, the use of the endodontic-restorative pertaining process provides the process for a proper protocol for root-filled teeth [38]. The outcomes and the location of the teeth have to be considered with more occlusal considerations to manage the complexity and challenges within the total system.

## Conclusions

In order for the endodontically treated teeth to remain in the oral cavity for the long term, the treatment must be successful in this regard. When it comes to minimally invasive endodontic treatment, the hydraulic qualities of bioceramic sealers help create a solid 3D bond, and the root canal is shaped to match the canal's morphology. All of these factors help make minimally invasive endodontic treatment more successful. Thus, endodontics may preserve teeth and their vital tissues in a variety of methods. The minimally invasive endodontic method may be achieved using a variety of techniques, including access opening, biomechanical preparation of canals, and surgical endodontics. The endodontic technique is very important for the development of the dentine system. Technological development and computer-based technology are necessary for the development of the dental treatment process. Technical developments allow dentists to monitor and diagnose the condition of teeth, and decide the drilling point and angle. In that case, root canal instrumentation improved due to technical development. Timely monitoring and assessing the status as well as stress on endodontic treated teeth with even numeric data also. This fosters a tooth restoration strategy, such as filling microbial leakage and restricting the infection from one tooth to another. Here, biotechnology plays a vital role to combine biology and technology to strengthen teeth's natural health, which can affect human healthy living aspects with oral health. 
